# Targeting Mitochondrial Oxidative Stress to Mitigate UV-Induced Skin Damage

**DOI:** 10.3389/fphar.2018.00920

**Published:** 2018-08-20

**Authors:** Rhonda M. Brand, Peter Wipf, Austin Durham, Michael W. Epperly, Joel S. Greenberger, Louis D. Falo

**Affiliations:** ^1^Department of Dermatology, University of Pittsburgh, Pittsburgh, PA, United States; ^2^Department of Medicine, University of Pittsburgh, Pittsburgh, PA, United States; ^3^Department of Chemistry, University of Pittsburgh, Pittsburgh, PA, United States; ^4^McGowan Institute for Regenerative Medicine, University of Pittsburgh, Pittsburgh, PA, United States; ^5^Department of Radiation Oncology, University of Pittsburgh, Pittsburgh, PA, United States; ^6^UPMC Hillman Cancer Center, University of Pittsburgh, Pittsburgh, PA, United States; ^7^Department of Bioengineering, University of Pittsburgh, Pittsburgh, PA, United States; ^8^Clinical and Translational Science Institute, University of Pittsburgh, Pittsburgh, PA, United States

**Keywords:** oxidative stress, mitochondria, photoaging, photocarcinogenesis, ROS

## Abstract

Unmitigated UV radiation (UVR) induces skin photoaging and multiple forms of cutaneous carcinoma by complex pathways that include those mediated by UV-induced reactive oxygen species (ROS). Upon UVR exposure, a cascade of events is induced that overwhelms the skin’s natural antioxidant defenses and results in DNA damage, intracellular lipid and protein peroxidation, and the dysregulation of pathways that modulate inflammatory and apoptotic responses. To this end, natural products with potent antioxidant properties have been developed to prevent, mitigate, or reverse this damage with varying degrees of success. Mitochondria are particularly susceptible to ROS and subsequent DNA damage as they are a major intracellular source of oxidants. Therefore, the development of mitochondrially targeted agents to mitigate mitochondrial oxidative stress and resulting DNA damage is a logical approach to prevent and treat UV-induced skin damage. We summarize evidence that some existing natural products may reduce mitochondrial oxidative stress and support for synthetically generated mitochondrial targeted cyclic nitroxides as potential alternatives for the prevention and mitigation of UVR-induced skin damage.

Ultraviolet radiation (UVR) is a primary risk factor for both melanoma and non-melanoma skin cancer, and the principal cause of photoaging. It is both a complete carcinogen and an immune suppressor. Of the three subcategories of UVR, only UVB (290–320 nm) and UVA (320–400 nm) reach the surface of the earth in substantial quantities and are thus responsible for most UVR-related health effects. While UVB typically comprises less than 10% of UVR, it is a high energy component that is mainly absorbed by epidermal cells, while UVA, the more prevalent component, is weaker but penetrates into the dermis (Valejo Coelho et al., 2016). Within the epidermis, keratinocytes and melanocytes are the major targets of UVR, while within the dermis UV-related damage to fibroblasts leads to collagen degradation and dysregulation of metalloproteinases and extracellular matrix homeostasis associated with photoaging. UVR absorbed from solar radiation can induce extensive skin damage through a variety of mechanisms ranging from direct DNA damage to those resulting from the oxidative stress caused by UVR induced reactive oxygen species (ROS). Like high energy radiation, UVR induces ROS that are strong oxidizers capable of reacting with biomolecules on a nano to millisecond time scale. When UVR-induced ROS overwhelms the skin’s natural antioxidant mechanisms, broad effects including indirect DNA damage, lipid and protein peroxidation, and immune dysregulation cumulate in cell dysfunction and death (Bohm and Hill, 2016; Brash, 2016; Damiani and Ullrich, 2016; Park and Kang, 2016; Piotrowska et al., 2016; Brem et al., 2017a; Mohania et al., 2017; Schuch et al., 2017; summarized in **Figure 1A**).

**FIGURE 1:**
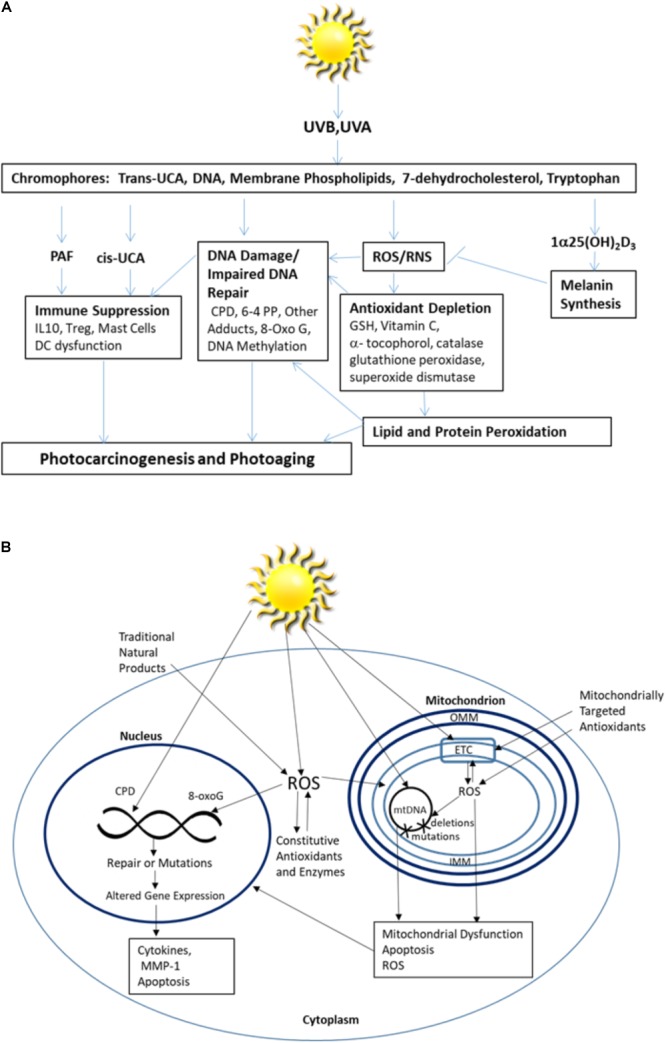
**(A)** Skin responses to excessive UV radiation and **(B)** the intracellular impact of excessive UV radiation.

## Endogenous Skin Protection Mechanisms Against UVR Damage

The skin has evolved multiple protective mechanisms to prevent extensive damage from UVR. Chromophores are a primary component of UVR skin protection pathways. They include a group of bioactive molecules, which absorb UVR energy through conformational and chemical changes, allowing them to serve as the first line of defense against UV radiation (Maddodi et al., 2012; Gibbs and Norval, 2013). When their radiation absorption capacity is overwhelmed, excess UVR leads to widespread cellular damage that includes damage to the chromophores themselves. Natural products capable of mitigating chromophore failure are often employed as UVR protective agents.

DNA is the most abundant chromophore present in the epidermis and the major chromophore implicated in UV-induced erythema (Young et al., 1998; Sklar et al., 2013). Excess absorption of UVR by DNA produces excited electron states and toxic-by products that initiate both direct and indirect DNA damage (Chen et al., 2014). UVB directly induces two types of DNA lesions; the more common cyclobutane pyrimidine dimers (CPD) are formed by two covalent bonds between the C5 and C6 carbon atoms of adjacent thymine (T) or cytosine (C) residues. In addition, 6-pyrimidine 4-pyrimidone photoproducts (6-4PP) are formed by one covalent bond between the 5′-end C6 and 3′ end C4 atoms of adjacent pyrimidines. Ideally, these photolesions are continuously removed at a rate sufficient to protect the cells. They may either be spontaneously resolved, targeted by the flavoenzyme, DNA photolyase, or by the nucleotide excision repair (NER) system (Yamamoto et al., 2017; Zhang et al., 2017).

Nucleotide excision repair is the primary DNA repair system capable of fixing these direct UVR-induced photolesions and is also critical for mending ROS-induced base modifications. DNA corrections by the NER pathway occurs through either transcription coupled repair (TCR) or global genome repair (GGR). In the former, lesions are specifically removed from the transcribed strand of active genes, while the latter system removes lesions throughout the genome (Britto et al., 2017). GGR begins as DNA damage sites are recognized by xeroderma pigmentosum C (XPC), are unwound by xeroderma pigmentosum B (XPB) and stabilized by the single stranded DNA binding proteins, xeroderma pigmentosum A (XPA), XPB, xeroderma pigmentosum D (XPD), and RPA. Repair is then initiated by DNA polymerase which uses the complementary strand as a template (Panich et al., 2016). When unresolved, these photolesions interfere with DNA transcription and replication leading to signature DNA mutations with C–T and CC–TT transitions and explain the high incidence of cancer in people diagnosed with xeroderma pigmentosum (Schuch et al., 2017) It has been suggested that sunscreens that contain DNA photolyase are superior to traditional sunscreens in at reducing field cancerization and CPD formation in patients with actinic keratosis (Carducci et al., 2015). In addition to serving as molecular triggers of skin cancer initiation (Kripke et al., 1992; Sreevidya et al., 2010), this DNA damage can lead to the release of immunosuppressive mediators (e.g., IL-10, TNF-α, and Vitamin D3) from keratinocytes and impaired function of cutaneous antigen presenting cells, that together result in UV-induced immunosuppression (Piotrowska et al., 2016; Prasad and Katiyar, 2017).

Pigment is also an important component of the chromophore protection system. Tanning is a photoprotective response to UV exposure that occurs through increased production of the pigment controlling chromophore melanin made by melanocytes. Melanin serves a protective role by absorbing and scattering UVR, converting the absorbed energy into heat, and limiting the UV-induced damage (Maddodi et al., 2012). However, when tryptophan and tyrosine absorb UVR, they can bind to DNA to produce photodamage and damage to adjacent amino acids within proteins (Maddodi et al., 2012). A photoproduct of tryptophan, the aryl hydrocarbon receptor agonist 6-formylindolo[3,2-*b*] carbazole (FICZ), acts as a UVA chromophore. Combined with UVA, FICZ generates ROS and induces oxidative DNA damage while inhibiting the removal of DNA photolesions by interfering with NER (Brem et al., 2017b). Importantly, keratinocytes can contribute to melanocyte protection by signaling through paracrine mediators, such as alpha-Melanocyte-stimulating-hormone (α-MSH), to mitigate cellular damage when exposed to excessive UVR (Jeayeng et al., 2017). α-MSH induces activation of NF-E2-related factor 2 (NRF-2), a well characterized transcription factor that serves as a master regulator of cellular antioxidant defense that induces expression of antioxidant enzymes including superoxide dismutase (SOD) 2, glutathione S-transferase, heme oxygenase 1, and NAD(P)H quinone dehydrogenase 1, in keratinocytes, melanocytes and fibroblasts (Gegotek and Skrzydlewska, 2015). Additionally, in the setting of excessive radiation exposure, NRF-2 transcriptionally activates nitric oxide synthases producing nitric oxide while reducing levels of oxidative radicals, such as superoxide and peroxynitrite (Xue et al., 2017).

Skin membrane phospholipids can also act as photoreceptors. A particularly relevant example is the production of platelet-activating factor (PAF) by keratinocytes upon UVR stimulation. UV-irradiation of keratinocytes activates the generation of ROS that promote the photoperoxidation of polyunsaturated cellular phospholipids. Oxidation of esterified fatty acyl residues introduces oxy functions, rearranges bonds and fragments carbon–carbon bonds by β-scission generating PAF and PAF-like lipids. PAF contributes to cancer progression by acting as a potent immune-suppressor, disrupting DNA repair, inducing apoptosis, and activating pro-tumorigenic epigenetic machinery (Damiani and Ullrich, 2016). PAF-R antagonists have been shown capable of preventing both skin cancer induction and progression (Sreevidya et al., 2008). In the stratum corneum, *trans* urocanic acid (*trans*-UCA) is produced by the action of L-histidine ammonia lyase on histidine. Upon UVR exposure, the chromophore *trans*-UCA undergoes a photoisomerization to its *cis*-isomer (Kaneko et al., 2008) (*Cis*-UCA). The conversion leads to intracellular ROS generation and oxidative DNA damage, PAF release and altered cytokine release, resulting in cell growth arrest and antigen presentation impairment (Gibbs and Norval, 2013) while simultaneously increasing protective [1α25(OH)_2_D_3_] vitamin D_3_ levels (Landeck et al., 2016). Similarly, UVB induces the conversion of the chromophore 7-dehydrocholesterol to vitamin D_3_ in keratinocytes. This biologically active form of vitamin D_3_ regulates epidermal differentiation, has anti-proliferative and immunosuppressive activity, and contributes to skin protection by increasing melanin synthesis (Piotrowska et al., 2016).

In addition to protective chromophores, the skin also constitutively expresses enzymatic and non-enzymatic antioxidants to defend against oxidative stress by preventing ROS generation, interfering with the free radicals generated, or removing damaged molecules before they accumulate to levels that can alter cell metabolism and viability. Within the skin, the epidermis contains higher concentrations of the major non-enzymatic antioxidants glutathione (GSH) and vitamin C, which are abundant cytosolic antioxidants, while α-tocopherol (vitamin E) is localized in cell membranes (Shindo et al., 1993; Godic et al., 2014). GSH is mainly present in the reduced form in the mitochondria and, while the concentration is similar to that of the cytosol (10–14 mM), it is only 10–15% of the total GHS pool (Marí et al., 2009). The primary constitutive enzymatic skin antioxidants include SOD, glutathione peroxidase and catalase all of which are induced by UVR exposure (Godic et al., 2014). SOD is divided into mitochondrially localized manganese superoxide dismutase (MnSOD) and cytosolic copper zinc superoxide dismutase (CuZn-SOD) (Azadmanesh and Borgstahl, 2018). While activity level varies by tissue type, MnSOD levels are consistently lower than CuZN-SOD (Janssen et al., 2000; Chiarelli et al., 2005). When the antioxidant system cannot remove sufficient ROS, excess ROS results in lipid peroxidation and oxidation of DNA and proteins (Terra et al., 2012). Oxidative damage to DNA is characterized by 8-Hydroxy-2′-deoxyguaninosine (8-OHdG), a DNA base-modified product associated with UVB-induced skin carcinogenesis (Wulff et al., 2008; Hirano, 2011; Gordon-Thomson et al., 2012). These oxidative DNA lesions are repaired via the base excision repair mechanism (BER), and failure of the system can lead to photocarcinogenesis (Valejo Coelho et al., 2016; Zinovkina, 2018). Without sufficient antioxidants to prevent the pro-oxidants that promote peroxidation, free radical induced lipid peroxidation disrupts membrane homeostasis and induces DNA damage (Hong et al., 2012). Similarly, oxidative damage to proteins including the formation of protein carbonyls, leads to disruption of normal cellular functions (Zhao and Robbins, 2009; Peres et al., 2011) and contributes to photoaging and carcinogenesis (Brem et al., 2017a).

## UVR-Induced Mitochondrial DNA Damage

Mitochondria are particularly sensitive to excessive UVR generated ROS, as they are a major source of cellular oxidants. Since mitochondrial DNA (mtDNA) is located in the matrix attached to the inner mitochondrial membrane, it is directly exposed to mitochondria generated ROS. mtDNA is very susceptible to damage as a result of a combination of factors, including the absence of introns that results in more than 95% coding sequences, the absence of protective histones, and few nucleotide excision repair mechanisms compared to nuclear DNA (Tulah and Birch-Machin, 2013).

UV radiation induced mitochondrial dysfunction directly contributes to photoaging (Naidoo et al., 2018). Even visible blue light (400–500 nm) contributes to photocarcinogenesis and photoaging by inducing chronic low levels of ROS in mitochondria (Nakashima et al., 2017). Mitochondrial susceptibility is magnified as damage to other cellular compartments is often coupled with increases in ROS in mitochondria (Frantz and Wipf, 2010). Accumulated damage to cellular signaling pathways generates chronically elevated levels of mitochondrial ROS, exceeding quantities that can typically be handled by intracellular antioxidants and accelerating mitochondrial damage. mtDNA is particularly susceptible to ROS induced damage. While ROS can induce mutations in both nuclear and mtDNA, UVR generates a 10-fold greater number of mutations in mitochondrial mtDNA than found in nuclear DNA (Naidoo et al., 2018). Further, mutations in mtDNA are more difficult to repair (Birch-Machin et al., 2013). mtDNA is deficient in protective mechanisms that ensure accurate replication, including histones, which proofread prior to replication, and the nucleotide excision repair pathway responsible for repairing UVR-induced photoproducts (Birch-Machin et al., 2013). ROS also act directly on unsaturated lipids, such as cardiolipin (CL), that are readily oxidized and converted into lipid second-messengers, propagating oxidative stress and disrupting mitochondrial bioenergetics (Kagan et al., 2009a; Liu et al., 2017). Mitochondrial dysfunction decreases ATP production and disrupts cellular calcium flux, which affects mitochondrial membrane potential and permeability in a feedback loop (Finkel et al., 2015). Under conditions of oxidative stress, cross-talk between mitochondria and the cell nucleus occurs through MnSOD and cyclin B1, which serve as checkpoint regulators (Herst et al., 2017; Kalen et al., 2017). This cross-talk influences tissue responses to UVR. Ultimately, activation of apoptotic and necrotic cell death pathways causes tissue dysfunction (Gaschler and Stockwell, 2017; summarized in **Figure 1B**). Given these increasingly apparent susceptibilities, it is not surprising that there is increasing interest in the development and identification of agents capable of preventing or mitigating ROS-induced mitochondrial damage. Strategies to protect mitochondria from UV induced ROS damage hold particular promise for the prevention and treatment of UVR-induced carcinogenesis and photoaging.

## Natural Antioxidants May Reduce Oxidative Stress in Mitochondria

Many ongoing efforts to develop UVR skin protectants leverage naturally occurring plant-derived compounds with potent antioxidant and anti-inflammatory properties. Their effectiveness in preventing UVR-induced skin damage in animal models and in clinical trials, by either oral or topical application, is the subject of considerable research and has been extensively reviewed (Vaughn et al., 2016; Addor, 2017; Cavinato et al., 2017; Lin et al., 2017; Samarth et al., 2017). These phytochemicals fall into multiple classes of chemicals including polyphenols such as the green tea extract epigallocatechin gallate (EGCG), resveratrol derived from grape skin, curcumin, and silymarin from milk thistle (Afaq and Katiyar, 2011; Bosch et al., 2015; Subedi et al., 2017); proanthocyanins such as those derived from grape seeds (Katiyar, 2016); and carotenoids including lycopene from tomatoes, β-carotene (Bosch et al., 2015), and others (Cavinato et al., 2017). Collectively, they have been observed to have both antioxidant and anti-inflammatory properties (Chen et al., 2012). These compounds have typically been evaluated as preventative agents, but in some cases their ability to mitigate radiation damage has been suggested (Brand and Jendrzejewski, 2008; Zhou et al., 2009; Delinasios et al., 2018).

Of the many natural products developed to prevent or mitigate UVR-induced skin damage, some have demonstrated a direct impact on mitochondrial function. Clinical trials with ingestion of the dietary carotenoid lycopene, derived from tomato paste, demonstrate protection from UVR that is associated with reductions in mtDNA 3895-bp deletions, and reduced expression of matrix metalloproteinase (MMP-1), an enzyme responsible for the degradation of the extracellular matrix proteins associated with photoaging (Rizwan et al., 2011). Lycopene appears to function through multiple mechanisms that include reduction of ROS, effects on cell signaling, and upregulation of endogenous antioxidant response element genes.

Polyphenols are the most common natural products that have been observed to improve the function of stressed mitochondria. Of the polyphenols, resveratrol has been the most extensively studied. Resveratrol has been shown to directly impact mitochondria by modulating the oxidative phosphorylation system. It can act as both a ROS scavenger by inducing gene expression of antioxidant enzymes such as MnSOD (de Oliveira et al., 2016), and by preventing UVR-induced apoptosis by inhibiting induction of p53, Bax, cleaved caspase activation and cytochrome C release (Subedi et al., 2017). Synthetic resveratrol analogs that improve stability, such as those that contain a butyrate or isobutyrate attachment or resveratrate, are more biologically active than the naturally occurring compounds and have demonstrated effectiveness in clinical trials after topical application (Wu et al., 2013; Lephart and Andrus, 2017). Both curcumin and grape seed extract protect skin against UVR by activating the mitochondrial protective Keap1-NRF-2 pathway (Ben Yehuda Greenwald et al., 2017; Che et al., 2017). Curcumin has been shown to improve skin diseases in multiple clinical trials when given either orally or topically (Vaughn et al., 2016). Glycyrrhizic acid, a bioactive triterpene, found in black licorice, also inhibits UVB-induced ROS and subsequent apoptosis by preventing pro-apoptotic Bax translocation into the mitochondria in cultured keratinocytes (Afnan et al., 2016). Melatonin and its metabolites have also been suggested to act as photoprotective agents through mitochondrial interactions (Abbaszadeh et al., 2017; Slominski et al., 2017).

Similarly, the polyphenol EGCG has been shown to protect mitochondria exposed to radiation. EGCG maintains mitochondrial mass and has been shown to have anti-apoptotic effects through suppression of pro-apoptotic Bax and inducing the anti-apoptotic BCL-2 proteins. Additionally, EGCG helps stabilize irradiated cells by inducing the mitochondrial antioxidant SOD2 (Zhu et al., 2014). EGCG is a main component of green tea, and multiple clinical trials have shown evidence of the cutaneous photoprotection from ingestion or topical application of green tea and its derivatives (Saric and Sivamani, 2016). Walnut seeds contain high levels of phenolic acids, including caffeic, chlorogenic, coumaric, ferulic, gallic, and tannic acids, that have been shown to protect keratinocytes from UVB-induced mitochondrial-mediated apoptosis by downregulating ROS through upregulation of an ROS eliminatory pathway (Park et al., 2014). Similarly, the flavonoid baicalin is a polyhydroxy phenol that acts as a free radical scavenger and has photodamage mitigating effects that are associated with preserved mitochondrial function (Farris et al., 2014; de Oliveira et al., 2015).

## Mitochondrially Targeted Synthetic Agents to Mitigate Oxidative Stress

**FIGURE 2:**
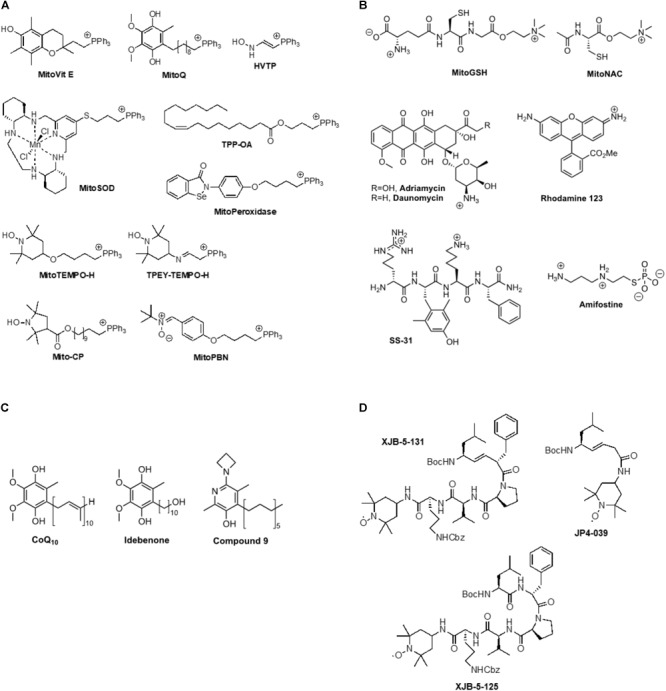
**(A)** Example for triphenylphosphonium-mediated transfer of antioxidants to mitochondria, **(B)** Ammonium cation-mediated transfer of antioxidants to mitochondria, **(C)** Lipophilic phenols, **(D)** Mitochondrially targeted nitroxides based on a gramicidin S peptide isostere sequence.

Achieving functionally effective concentrations of exogenous agents in mitochondria *in vivo* is a critical challenge to preserving mitochondrial function in the face of UVR induced oxidative stress. As an alternative to gene therapy approaches and biological therapeutics to counteract radiation damage, we and others have synthesized and assessed small organic molecules as radiation mitigators that have the potential to react with mitochondrial ROS and impede intrinsic apoptosis and necrotic cell death pathways (Hoye et al., 2008). An important approach to identifying early-response radiation mitigators is to develop pharmacophores that are able to neutralize cell death signals prior to CL oxidation, release of cytochrome c (Cyt c) into the cytosol, and activation of the executioner caspase 3 (Kagan et al., 2009b). An early strategy to attain high concentrations of antioxidants at the sites of oxidative stress involved their covalent attachment to lipophilic cations, such as a triphenylphosphonium group [(Ph)_3_P^+^; TPP]. This approach can enrich small molecular weight payloads in mitochondria up to 2000-fold over those found in the cytosol. Pertinent examples for this category include MitoVit E, MitoQ, HVTP, MitoSOD, MitoPeroxidase, MitoTempo, TPEY-TEMPO, Mito-CP, and MitoPBN (**Figure 2A**). The best characterized mitochondria-targeted antioxidant, MitoQ [(10-(4,5-dimethoxy-2-methyl-3,6-dioxo-1,4-cyclohexadien-1-yr)decyl)triphenylphosphonium], shuttles a ubiquinone moiety to mitochondria as a function of negative membrane potential and is effectively recycled to the active ubiquinol by complex II in the respiratory chain (Rodriguez-Cuenca et al., 2010). A similar approach was taken by using a mitochondrially targeted iron chelator to prevent UVA-induced labile iron-mediated oxidative damage as a protective measure against UVA-induced necrosis (Reelfs et al., 2016).

An intact mitochondrial membrane potential is also an important factor for the efficacy of positively charged ammonium species, such as MitoGSH, MitoNAC, adriamycin, daunomycin, rhodamine 123, SS-31, and amifostine (**Figure 2B**). The latter compound is a prodrug. The phosphate group is rapidly cleaved in solution, releasing a highly positively charged thiol that can scavenge a range of electrophilic species (Kouvaris et al., 2007). Intravenous amifostine has shown efficacy in a Phase III randomized trial in reducing the frequency of acute and chronic xerostomia in patients receiving radiation therapy for head and neck cancer (Radvansky et al., 2013). SS-31 (bendavia, elamipretide) has been shown to protect the structure of mitochondrial cristae and promote oxidative phosphorylation, and has thereby demonstrated efficacy in clinical trials of primary heart failure, mitochondrial myopathy, and Barth syndrome (Kagan et al., 2009b; Daubert et al., 2017).

Due to their lipophilicity, phenols such as CoQ_10_ and idebenone enrich in mitochondrial membranes where they exert classical antioxidant effects (**Figure 2C**; Fash et al., 2013). Compound 9 showed improved efficacy in quenching lipid peroxidation and ROS, preserving mitochondrial membrane potential and maintaining ATP synthesis (Alam et al., 2014).

The uncharged nitroxides XJB-5-131, JP4-039, and XJB-5-125 represent a promising class of mitochondrial targeting ROS, radical, and electron scavengers (**Figure 2D**; Hoye et al., 2008). These compounds were designed based on natural product lead structures to enrich in mitochondria. They neutralize free radicals and electrons escaping from the electron transfer chain by cycling from nitroxide to hydroxylamine, and, depending on the redox environment, to an oxoammonium species, thereby reducing ROS and counteracting oxidative stress generated after radiation exposure. XJB-5-131 was found to be approximately 600-fold enriched in mitochondria over the cytosol (Wipf et al., 2005; Jiang et al., 2007). This compound has shown *in vivo* efficacy in rodent models of Huntington’s disease (HD) (Xun et al., 2012; Budworth et al., 2015), traumatic brain injury (TBI) (Ji et al., 2012), ischemia-reperfusion injury (Ji et al., 2015; Jang et al., 2017), and hemorrhagic shock (Macias et al., 2007). In addition to being an effective anti-apoptotic agent, XJB-5-131 was also found to prevent ferroptotic cell death in HT-1080, BJeLR, and panc-1 cells (Krainz et al., 2016), revealing the critical role of intramitochondrial lipid peroxidation in ferroptosis. These results suggest that preventing mitochondrial lipid oxidation might become a viable therapeutic opportunity in pathologies that involve necrotic cell death pathways, including photoaging. The lower molecular weight analog, JP4-039, was able to prevent acute effects and mitigate chronic effects of radiation in mice (Epperly et al., 2017). Moreover, in mouse and human skin, topical application of JP4-039 prevented and mitigated radiation-induced skin damage characterized by clinical dermatitis, loss of barrier function, inflammation, and fibrosis. Damage mitigation was associated with reduced apoptosis, preservation of the skin’s antioxidant capacity, and reduction of irreversible DNA damage and protein degradation due to oxidative stress (Brand et al., 2017). Thus, targeted nitroxides such as JP4-039 have the potential to be used effectively in applications to protect normal tissue during clinical radiotherapy, to prevent and treat photoaging and photocarcinogenesis, and potentially as a countermeasure to mitigate damage after radiation exposure.

## Conclusion

Topically delivered and orally ingested natural products have been incorporated as preventative agents to delay skin photoaging and prevent UV-induced skin cancers with mixed success. Given the importance of UV-induced mitochondrial dysfunction in the development of both skin photoaging and skin cancer, it follows that agents capable of directly preventing this mitochondrial damage are more likely to be successful. To date, few natural products have been shown to directly reach mitochondria concentrations sufficient for protection. In this context, synthetic antioxidants, and in particular cyclic nitroxides, that can be designed to specifically localize in mitochondria have considerable promise. Early studies suggest that they may contribute significantly to developing strategies to reduce the incidence of UV induced skin cancers and prevent or delay photoaging.

## Author Contributions

RB, PW, AD, and LF wrote and/or edited a portion of the review article. ME and JG contributed to the concepts discussed.

## Conflict of Interest Statement

PW, ME, JG, and LF are inventors of related intellectual property covered by patents. The remaining authors declare that the research was conducted in the absence of any commercial or financial relationships that could be construed as a potential conflict of interest.
